# The accuracy of MRI in diagnosing and classifying acute traumatic multiple ligament knee injuries

**DOI:** 10.1186/s12891-021-04976-1

**Published:** 2022-01-13

**Authors:** Xusheng Li, Qian Hou, Xuehua Zhan, Long Chang, Xiaobing Ma, Haifeng Yuan

**Affiliations:** 1 Department of Spinal Orthopaedics, General Hospital of Ningxia Medical University, No. of 804, Shengli Street, Yinchuan, 750004 China; 2 Department of Radiology, General Hospital of Ningxia Medical University, No. of 804, Shengli Street, Yinchuan, 750004 China

**Keywords:** Multiple ligaments injuries, Knee dislocations, MRI, Value

## Abstract

**Background:**

Magnetic resonance imaging (MRI) is widely used for the evaluation of knee injuries, however, the accuracy of MRI in classifying multiple ligament knee injuries (MLKIs) remains unknown. This study aimed to investigate the accuracy of MRI in diagnosing and classifying acute traumatic MLKIs, we hypothesize that MRI had high accuracy in detecting and classifying MLKIs.

**Methods:**

The clinical data of 97 patients who were diagnosed with acute traumatic MLKIs and managed by multi-ligament reconstruction between 2012 and 2020 were retrospectively reviewed. The MR images were read by two experienced radiologists and results were compared with intraoperative findings, which were considered as the reference for the identification of injured structures. The value of MRI in detecting injuries of anterior cruciate ligament (ACL), posterior cruciate ligament (PCL), medial collateral ligament (MCL), lateral collateral ligament (LCL), and meniscus was evaluated by calculating the sensitivity, specificity, positive predictive value, negative predictive value, positive likelihood ratio, negative likelihood ratio, and kappa coefficients analysis. The value of MRI in classifying MLKIs was evaluated by calculating the agreement between MRI and intraoperative findings.

**Results:**

For detecting the specific injured structures in MLKIs, MRI had high sensitivity (90.7% for ACL, 90.4% for PCL, and moderate specificity (63.6% for ACL, 50% for PCL) in detecting cruciate ligament injuries, moderate sensitivity (79.1% for MCL, 55.6% for LCL) and specificity (46.7% for MCL, 68.4% for LCL) in detecting collateral ligament injuries, fair sensitivity (61.5%) and low specificity (39.4%) in the diagnosis of injuries to the meniscus. For classifying the MIKIs, MRI had a moderate agreement with intraoperative findings in classifying KD-V (kappa value = 0.57), poor agreement in the KD-I (kappa value = 0.39) and KD-IIIM (kappa value = 0.31), meaningless in the KD-II and KD-IIIL (kappa value < 0). The overall agreement between MRI and intraoperative findings in classifying MLKIs was poor (kappa value = 0.23).

**Conclusions:**

MRI is valuable in early detection and diagnosis of acute MLKIs, however, the accuracy of MRI in classifying MLKIs is limited. The management of MLKIs should be based on intraoperative findings, physical examinations, and comprehensive imaging results.

## Introduction

Multi-ligament knee injuries (MLKIs) are rare but serious injuries that are usually caused by high-energy trauma [1–3]. The definition of MLKIs is the complete tear of 2 or more cruciate and/or collateral ligaments, with or without injuries of meniscus, nerves, arteries, or periarticular fractures [4]. Some of the MLKIs have knee dislocations (KD), however, the dislocated knee can reduce spontaneously or have been reduced in the emergency department before hospitalization, thus the severity of the injured knee can be underestimated [1, 5, 6].

Early detection of injured structures is crucial for the management of MLKIs, MRI is the necessary preoperative imaging examination, which is also valuable in detecting nerve injuries [7]. The value of MRI for diagnosing isolated ligament injuries has been widely demonstrated, however, in terms of multi-ligament injuries, the accuracy of MRI is controversial. Derby et al. [8] found that MRI was sensitive in detecting injuries of cruciate and collateral ligaments, but not reliable in diagnosing injury to the meniscus or posterolateral corner (PLC). Twaddle et al. [9] demonstrated that MRI is not reliable for revealing injuries of the lateral collateral ligament (LCL) and PLC. However, Munshi et al. [10] reported that MRI had reliable sensitivity and specificity for detecting cruciate ligament injury and meniscal tears, even injuries that could not be precisely identified by arthroscopy. Similar results were found by Halinen et al. [11], and Kosy et al. [12]. In terms of reproducibility, Barbier et al. [13] demonstrated that MRI lacks precision and reproducibility, and the diagnosis should be integrated with clinical exam and stress X-rays. It has been also reported that MRI was inferior to clinical examination [14].

In short, the diagnostic value of MRI in MLKIs remains controversial, and the accuracy of MRI in classifying MLKIs has not been reported. This study aimed to investigate the accuracy of MRI in diagnosing and classifying acute traumatic MLKIs (within 3 weeks after injury). The intra-operative findings were considered the reference of injury patterns. We hypothesize that MRI had high accuracy in detecting and classifying MLKIs.

## Methods

### Patients

The clinical record database of knee surgeries in the orthopaedic department from one single center between 2012 and 2020 was retrospectively reviewed. Patients who were diagnosed with MLKIs and treated by multi-ligament reconstruction were included. The inclusion criteria were (1) Acute traumatic injury of at least two of the following ligaments: ACL, PCL, MCL, and LCL. (2) The 1.5 Tesla MRI was performed preoperatively and the images were available. (3) The injury patterns of knee structures were recorded in detail in the surgical notes and the injured structures were classified as strain, partial or complete tears. Since the injuries of nerve and vascular can not be fully revealed by intraoperative findings, preoperative MRI results were referenced. The exclusion criteria were (1) Revision of failed reconstructed ligaments. (2) Periarticular tumors, infections, or congenital disorders that were found during the surgery.

After admission, a standard 1.5-Tesla MRI was performed during the acute phase of the injuries. The MRI was performed using the Turbo Spin Echo (TSE) technique, The sequence parameters were: TE (20–100 ms), TR (3000–4000 ms), slice thickness (4.0 mm), spacing (0.5–1 mm), matrix (> 256*224), and FOV (180–230 mm). Perioperative X-Rays and CT scans were also performed to observe whether there were periarticular fractures and other lesions. Computed tomographic angiography (CTA) was performed to identify the injuries of arteries. All patients underwent a standard physical examination of the injured knees under anesthesia, the knee laxity was quantified by the stress test, knee arthrometer, and stress X-rays. The results were compared with the uninjured knee, and side-to-side differences were recorded. The reference standard was intraoperative findings. General information including age and gender was recorded.

### Evaluation of the diagnostic value of MRI

The MR images were analyzed by two experienced musculoskeletal radiologists who were blinded to the injury patterns independently to check the presence of injuries to the ACL, PCL, MCL, LCL, PLC, and meniscus. The MRI diagnosis was performed preoperatively, and the two radiologists were blinded to the MRI findings of each other. According to the integrity of the ligaments, the injured ligaments were classified as a partial tear (ligament was ruptured but was continuous, Grade 1 or 2) or a complete tear (interruption of ligament integrity, Grade 3), or avulsion of the ligament endpoints. The injured ligaments and meniscus were compared with intraoperative findings that were extracted from the surgical records. Disruption of periarticular bone tissues (including femoral condyle, tibial plateau, tibial intercondylar eminence, patella, and fibula head) was defined as periarticular fractures. The exclusion criteria were bone contusion, edema of bone marrow, and bony avulsion of ligaments. The reference standard of periarticular fractures was identified through preoperative X-ray, CT scan, and intraoperative findings either from an open or arthroscopic approach. The surgeries were performed by three chief surgeons, all patients underwent a single-stage reconstruction [15]. The ACL and PCL were reconstructed with autograft gracilis and semitendinosus. For MCL/LCL, a partial tear of MCL/LCL was sutured by non-absorbable wires; bony avulsions of the insertion were fixed with a suture anchor; ruptures in the mid-substance that cannot be repaired were reconstructed with a semitendinosus autograft.

The kappa statistic was used to determine the agreement between MRI and intraoperative findings. The diagnostic value of MRI was evaluated by calculating the sensitivity, specificity, positive predictive value (PPV), negative predictive value (NPV), positive likelihood ratio (PLR), negative likelihood ratio (NLR), accuracy, and kappa value. The accuracy was calculated by the (true positive + true negative)/( true positive + true negative + false positive + false negative). The sensitivity, specificity, and accuracy were defined as high (accuracy ≥ 85%), moderate (65%≤accuray<85%), fair (50%≤accuracy<65%), low (< 50%) [8]. The classification of MLKIs was based on the criteria that were described by Schenck et al. [16], both for the MRI results and the intraoperative findings. The results were compared and the agreement between them was evaluated by calculating the kappa value. The agreement was defined as good (*kappa* value ≥ 0.75); moderate (0.4 ≤ *kappa* value < 0.75), poor (*kappa* value < 0.4); meaningless (*kappa* value ≤ 0) [17].

### Statistical analysis

SPSS 25.0 (IBM Corp., Armonk, NY, USA) was used for statistical analyses. The normality of the quantitative data was checked by the Kolmogorov–Smirnov test. Normally distributed quantitative data were expressed by Mean ± Standard Deviation (*SD*), non-normal distributed quantitative data were presented as the interquartile range (*IQR*). Descriptive data were presented as numbers and percentages. The Kappa statistic was used to evaluate the agreement between the MRI and intraoperative findings, and the inter-rater reliability between the two radiologists for the MRI findings. Statistical significance was defined as *P* < 0.05.

## Results

### Study cohort

A total of 173 patients that were diagnosed with MLKIs were included for screening, 53 patients were excluded according to the criteria, the MRI results were not available in 23 patients. Finally, 97 patients (97 injured knees) were included for analysis. There were 69 males (71.1%) and 28 females (28.9%), the mean age was 41.3 (± 1.7) years. The combination of reconstructed ligaments and injured structures are described in Table 1. The MLKIs were classified as 20 KD-I (20.6%), 4 KD-II (4.1%), 47 KD-IIIM (48.5%), 5 KD-IIIL (5.2%), and 21 KD-V (21.6%) according to intraoperative findings (Table 2).

**Table 1:** The definition of classification and number of patients classified in each category using intra-operative findings

Grade	Definition	Number of patients
KD-I	Two ligaments ruptured, one cruciate ligament and one collateral ligament, ACL/PCL + MCL/LCL/PLC	20
KD-II	Two ligaments ruptured, both cruciate ligaments, ACL + PCL	4
KD-III	Three ligaments ruptured, both cruciate ligaments, and one collateral ligament, ACL + PCL + MCL (KD-IIIM), ACL + PCL + LCL/PLC (KD-IIIL)	52
KD-IV	Both two cruciate ligaments and two collateral ligaments ruptured, ACL + PCL + MCL + LCL/PLC	0
KD-V	Any KD classifications that were accompanied by periarticular fractures or knee dislocations	21

**Table 2:** Demographic characteristics and the combined injuries of the study cohort

Reconstructed ligaments	n	Age (year, mean ± *SD*)	Male/Female (n)	Meniscus injury (n)	Artery injury (n)	Nerves injury (n)	Patellar dislocation (n)	Periarticular fracture (n)
ACL + MCL	15	38.2 ± 8.5	9/6	5	1	-	2	5
ACL + LCL	1	45	0/1	-	-	-	-	-
ACL + PLC^*^	2	39.5 ± 2.1	1/1	-	-	-	-	1
PCL + MCL	3	43.7 ± 9.1	1/2	-	-	-	-	-
PCL + LCL	2	48.5 ± 3.5	2/0	-	-	-	-	1
PCL + PLC	6	41.5 ± 16.1	6/0	1	-	-	-	1
ACL + PCL	8	41.7 ± 14.0	6/2	7	2	1	1	3
ACL + PCL + MCL	52	43.9 ± 11.9	37/15	12	-	2	7	8
ACL + PCL + LCL	7	41.7 ± 16.0	7/0	3	1	1	-	2
ACL + PCL + PLC	1	57	1/0	-	1	-	-	-
Entire cohort	97	42.6 ± 11.8	70/27	28	5	4	10	21

*SD* standard deviation

^−^The number was zero

### Diagnostic value of MRI

**Fig. 1:**
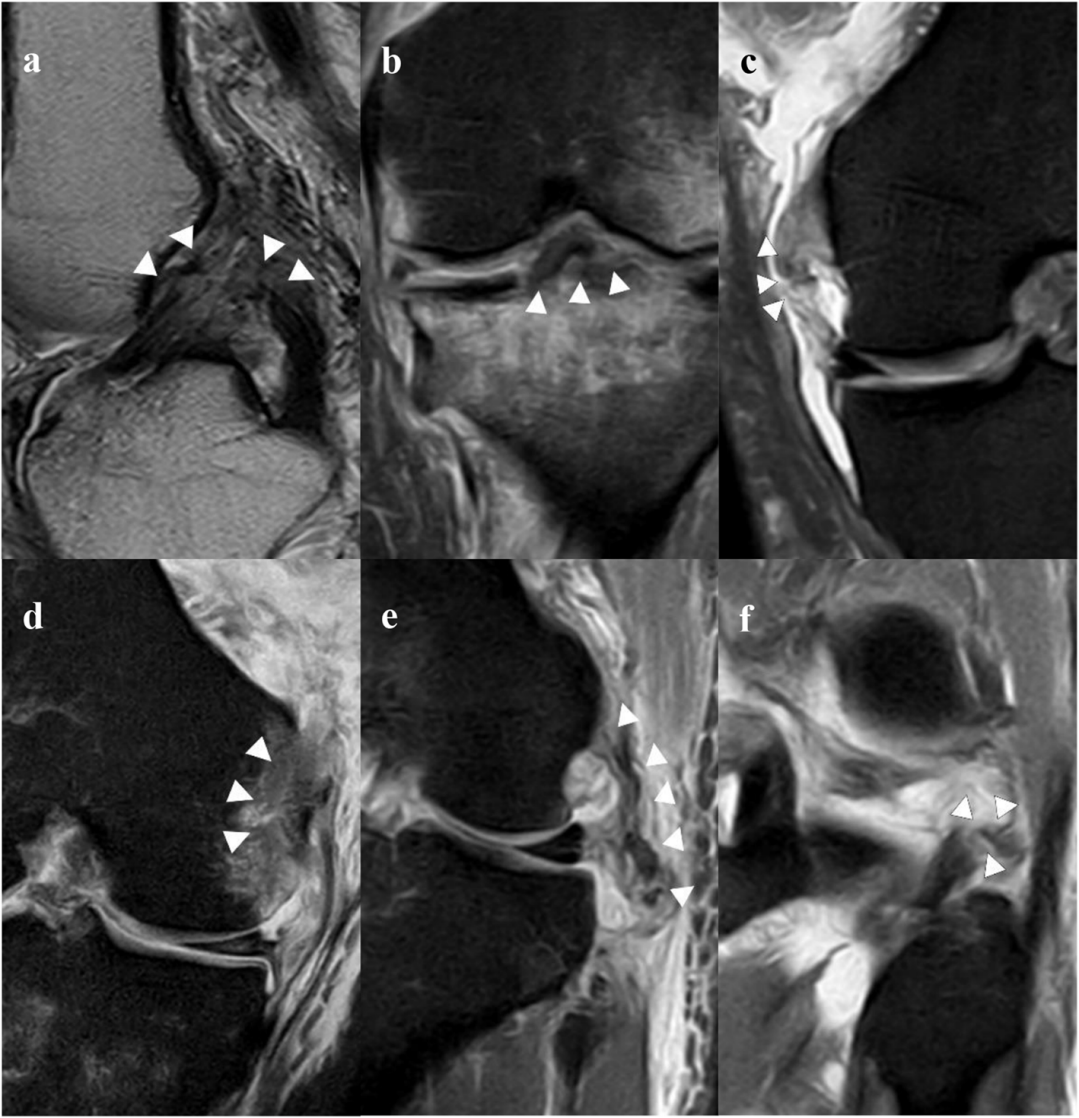
Preoperative MRI of the multiple ligaments knee injuries/knee dislocations; **a** The ruptured ACL and PCL; **b** Avulsion fracture at the ACL insertion in the tibial intercondylar eminence; **c** Rupture of MCL in the mid-substance; **d** Avulsion of MCL at the insertion of the femoral condyle; **e** The ruptured LCL in the mid-substance; **f** The ruptured popliteal tendon

The overall agreement across all MRI results between the two radiologists was good (*kappa* = 0.83, *P* < 0.001). MRI was found to have high sensitivity (90.7%) and moderate specificity (63.6%) in the diagnosis of injuries to the ACL; high sensitivity (90.4%) and moderate specificity (50%) in the diagnosis of injuries to the PCL; moderate sensitivity (79.1%) and low specificity (46.7%) in the diagnosis of injuries to the MCL; fair sensitivity (55.6%) and moderate specificity (68.4%) in the diagnosis of injuries to the LCL; fair sensitivity (61.5%) and low specificity (39.4%) in the diagnosis of injuries to the meniscus. The accuracy was good for ACL injuries (87.6%) and PCL injuries (84.5%), moderate for MCL injuries (69.1%) and LCL injuries (66.9%), low for the meniscus injuries (45.4%). The agreement between MRI results and intraoperative findings was moderate in the ACL injuries (*kappa* = 0.47) and PCL injuries (*kappa* = 0.39), poor in the MCL injuries (*kappa* = 0.26) and LCL injuries (*kappa* = 0.18), and meaningless in tears of the meniscus (Table 3). Only one of the 9 injured PLC was revealed by preoperative MRI, so the sensitivity and specificity cannot be calculated. The preoperative MR images of the injured ligaments are shown in Fig. 1.

**Table 3:** The diagnostic value of MRI in the MLKIs

Structure	Sensitivity (%)	Specificity (%)	PPV (%)	NPV (%)	PLR	NLR	Accuracy (%)	Kappa value^a^	*P* value^b^
ACL	90.7	63.6	95.1	46.7	2.5	0.2	87.6	0.47	< 0.001
PCL	90.4	50	91.5	46.7	1.8	0.2	84.5	0.39	< 0.001
MCL	79.1	46.7	76.8	50	1.5	0.5	69.1	0.26	0.010
LCL	55.6	68.4	28.6	87.1	1.8	0.7	65.9	0.18	0.057
Meniscus	61.5	39.4	27.1	73.7	1.0	0.9	45.4	0.01	0.931

*PPV* positive predictive value; *NPV* negative predict value; *PLR* positive likelihood ratio; *NLR* negative likelihood ratio

^*a*^The agreement was good (kappa value ≥ 0.75); moderate (0.4 ≤ kappa value < 0.75); poor (kappa value < 0.4); meaningless (kappa value < 0)

^*b*^If *p* < 0.05, the agreement is significant

Of the 97 combined injuries, 7 could not be classified by preoperative MRI findings. The classification based on MRI results and intraoperative findings was consistent in 51 patients (52.6%), of them, there were 13 KD-I (25.4%), 3 KD-II (5.9%), 22 KD-IIIM (43.1%), 1 KD-IIIL (1.9%), and 12 KD-V (23.5%). The kappa statistics showed that MRI has a moderate agreement with intraoperative findings in classifying KD-V (*kappa* value = 0.57), poor agreement in the KD-I (*kappa* value = 0.39) and KD-IIIM (*kappa* value = 0.31), while meaningless in the KD-II and KD-IIIL (*kappa* value < 0). The overall agreement in classifying MLKIs was poor (*kappa* value = 0.23) (Table 4).

**Table 4:** The value of MRI in classifying MLKIs according to Schenck classification (n)

Intraoperative classification (n)	MRI classification (n)							Total (n)	Kappa value^#^	*P* value^&^
	I	II	IIIM	IIIL	IV	V	NA*			
I	**13**	-	1	1	4	-	1	20	0.39	0.019
II	2	**3**	2	1	2	-	-	10	-0.07	0.619
IIIM	1	8	**22**	4	6	-	3	44	0.31	0.003
IIIL	-	4	1	**1**	1	-	1	8	-0.21	0.262
IV	-	-	-	-	-	-	-	-	-	-
V	-	-	-	1	-	**12**	2	15	0.57	0.007
Total	16	15	26	8	13	12	7	97	0.23	0.001

^*^NA Cannot be classified by Schenck classification

^#^The agreement was good (kappa value ≥ 0.75); moderate (0.4 ≤ kappa value < 0.75); poor (kappa value < 0.4); meaningless (kappa value < 0)

^*&*^If *p* < 0.05, the agreement is significant

^−^The number was zero

## Discussion

The present study found that the accuracy of MRI was good for detecting cruciate ligament injuries, moderate for collateral ligament injuries, low for meniscus injuries. We reviewed the literature and did not find research that investigated the value of MRI in classifying MLKIs. Though MRI performs better in classifying KD-V MLKIs, the overall agreement with intraoperative findings was poor. In short, MRI helps early detection of MLKIs, however, it has limited value in classifying the MLKIs preoperatively. The management of MLKIs should be based on comprehensive assessment including preoperative imaging, physical exam, and intraoperative exploration.

The diagnostic value of MRI in detecting multi-ligament injuries was evaluated by comparing the MRI results with clinical examination and/or intraoperative findings, but the results differ a lot [9–11, 14]. In most studies, MRI was reliable in detecting ligament injuries, however, in terms of meniscus and PLC, the conclusions were controversial [7, 8]. In the present study, the accuracy of MRI in detecting cruciate ligaments was consistent with previous studies. The accuracy was demonstrated moderate in detecting injuries to collateral ligaments, which was rarely reported in MLKIs. Research has suggested that oblique coronal and oblique sagittal MRI, which was parallel to the long axis of the ACL, improved the accuracy of the diagnosis of an ACL tear and the grading of ACL injury [18–20]. However, the application of oblique MRI in multiple ligaments injuries is limited. The evaluation of the value of MRI in diagnosing MLKIs should also consider the interpretation of MRI results [21]. Since the MLKIs were complex injuries, the accuracy of MRI for diagnosing isolated ligament injuries was not comparable with that of the multi-ligament injuries. In short, this study concluded that MRI was valuable for the early diagnosis of MLKIs.

The value of MRI in classifying MLKIs according to Schenck classification was explored in this study. We find that MRI has a moderate agreement in classifying KD-V, poor agreement in classifying KD-I and KD-IIIM, meaningless in KD-II and KD- IIIL. We speculate that the meaningless agreements in the KD-II and KD-IIIL were due to the small numbers of those two injuries, the diagnostic value cannot be reflected well. Though inferior to the CT scan, the present study revealed that MRI helps detect periarticular fractures (moderate consistency with intraoperative findings in classifying KD-V). Besides, we found that MRI has high sensitivity in detecting ACL and PCL injuries, but the overall agreement was poor compared to intraoperative findings. The results were not surprising because the MLKIs are complex injuries, a precise MRI-based classification is challengeable. Though the sensitivity and specificity in this study differ from previous studies, we concluded MRI has limited value in classifying MLKIs preoperatively, the management of MLKIs should be based on a comprehensive evaluation, including physical exam, combined X-Rays, CT, and mechanisms of injuries until intraoperative evidence was obtained.

In the present study, only one of the PLC injuries was revealed by preoperative MRI, suggesting a limited value of MRI in detecting PLC injuries, there were no false-positive cases, the sensitivity and specificity were not calculated because the number of samples was small. In fact, few studies have reported the results of PLC reconstruction because of the low incidence rate. Derby et al. [8] investigated the value of MRI in detecting the PLC for patients with knee dislocations, including LCL (76% accuracy, 100% sensitivity, 67% specificity) and iliotibial tract (89% accuracy, 97% specificity). In the present study, only 9 cases were diagnosed with PLC injuries according to the intraoperative findings. Precise detection of PLC injuries using MRI is challenging. The value of MRI in detecting PLC injuries remains unknown, the diagnosis should be based on clinical examination under anesthesia and intraoperative findings.

The present study has some limitations. First, this is a retrospective analysis with a small number of samples, there was a lack of systematic methods used to include/exclude patients, thus inherent bias can not be avoided. Second, there was a variation of the incidence of injury patterns between groups, the intraoperative findings were considered as gold standard during the statistical analysis, which may lead to bias. Third, only a 1.5 T MRI magnet was used for scanning and the severity of the injured ligaments was not graded using the MR images, partial and complete tears were not divided into subgroups and evaluated. Fourth, the number of PLC injuries was small and only one of them was successfully revealed by the MRI, thus the accuracy of MRI in detecting PLC injuries can not be evaluated. Furthermore, MRI results were not compared with clinical examination. Future studies should be based on larger samples and a more specific evaluation system.

## Conclusions

This study revealed that MRI has high sensitivity but moderate specificity in detecting ACL and PCL injuries, and a fair agreement in classifying KD-V MLKIs. In short, MRI was found to have limited value in classifying MLKIs. Therefore, the management of MLKIs should be based on intraoperative findings, physical examinations, and comprehensive imaging results.

## Data Availability

All data generated or analyzed during this study are included in this published article.
